# Supplementary material for: Patterns of Intron Gain and Loss in Fungi
Source: PLoS Biol. 2004 Nov 30;2(12):e422. doi: 10.1371/journal.pbio.0020422 (PMC532390; doi:10.1371/journal.pbio.0020422)
Supplement: Table S1 — Also available at http://genes.mit.edu/NielsenEtAl/. (4.3 MB ZIP). [file pbio.0020422.st001.zip › NielsenEtAl/html/632.html]

AN8782.1.NCU00173.1.MG05317.1.FG06681.1


```
 CLUSTAL W (1.82) Multiple Sequence Alignments - Introns Inserted


Sequence 1: AN8782.1	288 aa
Sequence 2: NCU00173.1	295 aa
Sequence 3: MG05317.1	287 aa
Sequence 4: FG06681.1	285 aa
Alignment Length: 296 aa
Number Identitical Residues: 150 aa
Alignment Score (without introns) 7574


MG05317.1 	MAFTTKATIASFGGRLLKLSHQSSVTGTEMAVNLYLPPQANKQ---KVPLLFYLSGLTCS
NCU00173.1	MGLTVKATIASFGGKLFKLSHPSTSTGTDMAVNLYLPPQALKSQT-KVPVLFYLSGLTCT
FG06681.1 	MAFTTNATINSFGGRFLKLSHKSAVTGTPMNATLYLPGSASSSKP--APLLVYLSGLTCT
AN8782.1  	MSVTTKATIASFGGKLLKLSHAAKSTNCEMAFNLYLPPQAYKTPSQKLPLLIYLSGLTCT
          	*..*.:*** ****:::**** :  *.  *  .**** .* .  ... *:*.*******:

MG05317.1 	PENCTEKGFFQHGASKHGIAVAYPDTSPR~GLGLPGEDESWDFGSAASFYVDAKQDPWKG
NCU00173.1	PDNCSEKGFFQHGASQRGLAIVYPDTSPR~GLNLPGEKDAWDFGEGAGFYVDATQEPWAK
FG06681.1 	PDNVTEKGFLHAHASKLGLALLYPDTSPR~ETDLPGEHDSYDFGSGAGFYIDAKKDPYSQ
AN8782.1  	GDNCSEKGFFQHGASKKGIAVLYPDTSPR1GLNIPGENDTWDFGTGAGFYVNATKEPYNK
          	 :* :****::  **: *:*: *******   .:***.:::*** .*.**::*.::*:  

MG05317.1 	NYRMETYITEELPRLLYEGFADKLD--KAKGMSITGHSMGGHGALSLYLKHPAMYRSVSA
NCU00173.1	GYKMDTYITRELPSVLFGADSEFGKYLDADKVSITGHSMGGHGALTLYLKNPGMYKSVSA
FG06681.1 	HYKMETYITKELPDLIFKEFKEV----DSSRVSISGHSMGGHGALTLFLKNPGMYKSVSA
AN8782.1  	GYNMYTYITEELPATVFEAFPQI----DSSRVSITGHSMGGHGALTLFLRNPGKYKSVSA
          	 *.* ****.***  ::    :     .:. :**:**********:*:*::*. *:****

MG05317.1 	FAPIANPSKCPWGEKAFGGYLGDD-RDEWKKHDSTELVKG--WNKDQDLKMLVDV~GTGD
NCU00173.1	FAPIANPCECAWGKKAFAGYLGEENKEEWKKHDATELIKSEKWRGHKDARVLVDV~GTGD
FG06681.1 	WAPISNPSQCPWGEKAFKGYLGED-KEEWKKHDATELIKS--WKG--KFPALIDV0GTGD
AN8782.1  	FAPISNPINCAWGQKAFGGYLGEENKEKWKEHDATELVKG--WAG-KNVDILIDV0GTGD
          	:***:** :*.**:*** ****::.:::**:**:***:*.  *   ..   *:** ****

MG05317.1 	NFYKQGQLLPENLEKAVKDAGLKGLTLRYQE0DYDHSYFFMAS1FSDDHVAHAAKYLDI-
NCU00173.1	NFYKQGQLLPENFEKAVKEAGVEGVTVRYQE0DYDHSYYFMAS~FSDEHVDHAAKYLGLL
FG06681.1 	NFYKQNQLLPENLEKAVKDAGIDGMTLRYQD0GYDHSYFFIST~FGEDHVKHAAKALGLL
AN8782.1  	NFYKQGQLLPENLEKAAKEAGVTGINVRYQP~DYDHSYYTMAT~FSDDHVEHAAKYLFA-
          	*****.******:***.*:**: *:.:***  .*****: ::: *.::** **** *   

AN8782.1  	
NCU00173.1	
MG05317.1 	
FG06681.1 	
          	
```
